# Health‐related quality of life in dogs treated with electrochemotherapy and/or interleukin‐12 gene electrotransfer

**DOI:** 10.1002/vms3.232

**Published:** 2020-01-07

**Authors:** Nina Milevoj, Natasa Tozon, Sabina Licen, Ursa Lampreht Tratar, Gregor Sersa, Maja Cemazar

**Affiliations:** ^1^ Veterinary Faculty Small Animal Clinic University of Ljubljana Ljubljana Slovenia; ^2^ Faculty of Health Sciences University of Primorska Isola Slovenia; ^3^ Institute of Oncology Ljubljana Ljubljana Slovenia

**Keywords:** dogs, electrochemotherapy, gene electrotransfer, health‐related quality of life, interleukin‐12

## Abstract

The aim of this study was to evaluate the owners' perception of health‐related quality of life (HRQoL) of dogs after treatment with electrochemotherapy (ECT) alone or combined with interleukin‐12 gene electrotransfer (IL‐12 GET) and/or surgery. The owners of 44 dogs with histologically different tumours were offered the »Cancer Treatment Form« at least one month after treatment. The owners assessed their dogs’ quality of life (QoL) after treatment as good (mean 7.4) (from 1–very poor to 10–excellent) and the general health compared with the initial diagnosis of cancer as improving (mean 3.9) (from 1–worse to 5–better). The assessment of the current QoL was better within the group of dogs treated with non‐invasive treatment (ECT and/or IL‐12 GET only), compared with those that received invasive treatment, where, in addition to ECT and/or IL‐12 GET, surgery was performed (*p* < .05). The owners of dogs that achieved an objective response (OR) to the treatment assessed the QoL as significantly better compared with those whose dogs did not respond to the treatment (*p* < .05). The majority of the owners (86.4%) would opt for the therapy again, regardless of the financial costs. In conclusion, the results of this study demonstrate that the majority of the owners of dogs assessed their dogs’ QoL as good and felt that it improved after the treatment, especially in dogs, treated with non‐invasive treatment and in those that responded to the treatment**.** This supports further use of ECT and IL‐12 GET as suitable methods for the treatment of selected tumours in veterinary medicine.

## Graphical abstract

The aim of this study was to evaluate the owners' perception of health‐related quality of life (HRQoL) of dogs after treatment with electrochemotherapy (ECT) alone or combined with interleukin‐12 gene electrotransfer (IL‐12 GET) and/or surgery. The results of this study demonstrate that the majority of the owners of dogs assessed their dogs’ quality of life as good and felt that it improved after the treatment, especially in dogs, treated with non‐invasive treatment and in those that responded to the treatment.

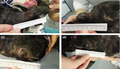

## 1 INTRODUCTION

There is no general consensus on the definition of quality of life (QoL) in veterinary medicine (Belshaw & Yeates, 2018). It has many different proposed definitions, but broadly represents the aspects of an animal's life that make life better or worse for that specific animal (Belshaw & Yeates, 2018). Health‐related quality of life (HRQoL) is a narrower term, defining the specific effect of a medical condition on an individual's health (Cella, 1992). In oncology, HRQoL defines the effect of cancer and its treatment on body function and well‐being (Lynch, Savary‐Bataille, Leeuw, & Argyle, 2011). Information about HRQoL can help making treatment decisions, provide prognostic information, improve owner–veterinarian interaction and evaluate the impact of new treatments in clinical trials (Lynch et al., 2011; Bottomley et al., 2005).

In human medicine, QoL is considered one of the main goals and an important decision‐making factor in clinical trials and cancer treatment. However, in veterinary medicine, this field has only begun to gain importance in the past two decades. Several questionnaires have been proposed for assessing QoL of dogs with chronic pain (Belshaw & Yeates, 2018), neurological and musculoskeletal pain (Silva, Luna, Joaquim, Coutinho, & Possebon, 2017), spinal cord injuries (Budke et al., 2008), cardiac disease (Freeman, Rush, Farabaugh, & Must, 2005; Oyama et al., 2008), obesity (Yam et al., 2016), diabetes mellitus (Niessen et al., 2012), epilepsy (Packer & Volk, 2015; Wessmann, Volk, Packer, Ortega, & Anderson, 2016), skin disease (Noli et al., 2011; Noli, Minafò, & Galzerano, 2011), portosystemic shunt (Greenhalgh et al., 2014) and in cats with chronic kidney disease (Bijsmans, Jepson, Syme, Elliott, & Niessen, 2016), cardiac disease (Freeman et al., 2012; Reynolds et al., 2010) and skin disease (Noli, Borio, Varina, & Schievano, 2016). In veterinary oncology, QoL was evaluated in dogs with mammary tumors (Faustino & Lallo, 2015) and in animals with different types of tumors (Lynch et al., 2011), treated with chemotherapy (Iliopoulou, Kitchell, & Yuzbasiyan‐Gurkan, 2013; Mellanby, Herrtage, & Dobson, 2003; Thornton, Cave, Bridges, & Stell, 2018; Tzannes, Hammond, Murphy, Sparkes, & Blackwood, 2008; Vøls, Heden, Kristensen, & Sandøe, 2017), tyrosine kinase inhibitors (London et al., 2009) and radiation therapy (Hill et al., 2014).

Electrochemotherapy (ECT) combines the use of chemotherapy and electroporation to achieve local tumour control (Tozon et al., 2016). Gene electrotransfer (GET), also known as electro‐gene transfer (EGT), is used in oncology to enhance the antitumor immune response (Impellizeri, Aurisicchio, Forde, & Soden, 2016). One of the most promising is GET of plasmid encoding interleukin‐12 (IL‐12), which has local and systemic effects on various tumors (Cemazar, Jarm, & Sersa, 2010). ECT alone, or combined with IL‐12 GET, is efficient in treating selected tumours in cats and dogs with minimal or no side effects (Cemazar et al., 2017; Kodre et al., 2009; Lowe, Gavazza, Impellizeri, Soden, & Lubas, 2017; Milevoj et al., 2019; Salvadori et al., 2017; Tozon, Kodre, Sersa, & Cemazar, 2005; Tozon, Pavlin, Sersa, Dolinsek, & Cemazar, 2014). In human medicine, QoL has been assessed for patients undergoing irreversible electroporation (Scheltema et al., 2018) and electrochemotherapy (Campana, Testori, Curatolo, & Quaglino, 2016). However, QoL has not been assessed for veterinary patients undergoing electroporation‐based treatments, such as ECT or GET. Therefore, the purpose of this study was to evaluate the owners' perception of HRQoL of dogs, treated with ECT alone or in combination with IL‐12 GET and/or surgery.

## 2 METHODS

The owners of dogs with histologically different tumours, treated with ECT alone or in combination with IL‐12 GET and/or surgery, were offered the »Cancer Treatment Form« at least one month after treatment*.*

At the time of the treatment, each tumour nodule was measured in three perpendicular directions (*a*, *b* and *c*) and the tumour volume calculated using the following formula: *V* = *a* × *b* × *c* × *π* ÷ 6. One month after treatment, the response to the treatment was evaluated according to the RECIST criteria (Nguyen, Thamm, Vail, & London, 2015).

The HRQoL_slo_ questionnaire (»Cancer Treatment Form«) was adapted from the questionnaire for assessing HRQoL in dogs and cats with cancer, developed by Lynch et al., 2011). It is composed of 23 questions, divided in eight domains (happiness, mental status, pain, appetite, general appearance, hydration, mobility and general health) and designed to assess the physical and physiological aspects of the animal's health. Each domain had three statements scored on a scale from 1 (strongly disagree) to 5 (strongly agree). The final question was a Visual Assessment Scale (VAS) evaluation of current QoL ranging from 1 (very poor) to 10 (excellent) and providing an assessment of HRQoL on a continuum. Health was assessed as‐it‐was at the time the questionnaire was completed (from 1‐worse, 3‐the same to 5‐better), compared with the health status before treatment. Four questions addressing wound care after treatment and treatment costs were added to the original questionnaire. The Slovenian version of the questionnaire was prepared based on content validity established by the veterinary and oncology experts. The internal reliability of the questionnaire was acceptable, with the Cronbach *α* = 0.6712.

For the purposes of data processing, the basic descriptive statistics were used as well as a non‐parametric Mann–Whitney U test to find out the statistically significant differences between the dogs’ current HRQoL and the type of treatment and the assessment of treatment outcomes (Nguyen et al., 2015). In addition, a linear regression analysis was applied. *p* ≤ .05 were considered significant.

## 3 RESULTS

From October 2017 to April 2019, 44 dogs of different breeds with histologically different tumours were included in the prospective, non‐randomized study. The age of dogs ranged from 5 to 16 years (median 9.0 years). A total of 29/44 (65.9%) of dogs were females and 15/44 (34.1%) were males. The most common breeds were crossbreed (18.2%), German Boxer (6.8%) and American Staffordshire Terrier (6.8%). The majority of dogs were diagnosed with mast cell tumours (68.2%), followed by oral malignant melanomas (9.1%), neurofibrosarcomas (4.5%), oral fibrosarcomas (4.5%), plasma cell tumours (4.5%) and other tumour types (9.2%). The volumes of the tumours ranged from 0 cm^3^ (residual microscopic disease) to 64.1 cm^3^ (median 2.05 cm^3^). The dogs were divided into two treatment groups. In the non‐invasive treatment group, only ECT and/or IL‐12 GET was performed. When ECT and/or IL‐12 GET was used in combination with surgery, dogs were assigned to the invasive treatment group. There were 33/44 (75%) of animals in the non‐invasive treatment group and 11/44 (25%) in the invasive treatment group. The demographic data and grouping are presented in Table 1.

**Table 1:** Demographic data and treatment groups

Gender		Age (years)		
Male	Female	6–8	9–12	13–16
15 (34.1%)	29 (65.9%)	19 (43.3%)	18 (40.9%)	7 (15.9%)
			*n*	%
Breed				
Crossbreed			8	18.2
German Boxer			3	6.8
American Staffordshire Terrier			3	6.8
Beagle			2	4.5
West Highland White Terrier			2	4.5
Cocker Spaniel			2	4.5
French Bulldog			2	4.5
Greyhound			2	4.5
Boston Terrier			2	4.5
Miniature Schnauzer			1	2.3
Bull Terrier			1	2.3
Jack Russell Terrier			1	2.3
Jagdterrier			1	2.3
Berger Blanc Suisse			1	2.3
Pug			1	2.3
Golden Retriever			1	2.3
Maltese Dog			1	2.3
English Setter			1	2.3
Bloodhound			1	2.3
Bernese Mountain Dog			1	2.3
Flat‐Coated Retriever			1	2.3
Staffordshire Bull Terrier			1	2.3
Alaskan Malamute			1	2.3
Shetland Sheepdog			1	2.3
Soft Coated Wheaten Terrier			1	2.3
Basset Hound			1	2.3
Weimeraner			1	2.3
Type of tumour				
Mast cell tumour			30	68.2
Oral malignant melanoma			4	9.1
Plasma cell tumour			2	4.5
Oral fibrosarcoma			2	4.5
Neurofibrosarcoma			2	4.5
Soft tissue sarcoma			1	2.3
Histiocytic sarcoma			1	2.3
Squamous cell carcinoma			1	2.3
Fibrosarcoma			1	2.3
Type of treatment				
ECT + GET			27	61.4
Surgery + ECT + GET			8	18.2
GET			4	9.1
ECT			2	4.5
Surgery + GET			2	4.5
Surgery + ECT			1	2.3

In the general health assessment section, owners were asked to evaluate the changes in their pets’ health after the treatment, based on a scale from 1 (strongly disagree) to 5 (strongly agree) (Table 2).

**Table 2:** Assessment of the “Cancer treatment form”

Domain	Item	Min	Max	Mean	*SD*
Happiness	My pet wants to play	2	5	4.5	0.9
	My pet responds to my presence	4	5	5.0	0.2
	My pet enjoys life	3	5	4.8	0.5
Mental status	My pet has more good days than bad days	3	5	4.7	0.6
	My pet sleeps more, is less awake	1	5	2.8	1.4
	My pet seems dull or depressed, not alert	1	5	1.4	0.9
Pain	My pet is in pain	1	5	1.9	1.2
	My pet pants frequently, even at rest	1	5	1.5	1.1
	My pet shakes or trembles occasionally	1	5	2.0	1.4
Appetite	My pet eats the usual amount of food	2	5	4.7	0.7
	My pet acts nauseated or vomits	1	4	1.6	0.8
	My pet eats treats/snacks	1	5	4.3	1.2
Hygiene	My pet keeps itself clean	1	5	1.4	1.0
	My pet smells like urine or has skin irritation	1	3	1.1	0.3
	My pet's hair is greasy, matted, rough	1	3	1.1	0.4
Water intake	My pet drinks adequately	4	5	4.9	0.3
	My pet has diarrhoea	1	5	2.1	1.3
	My pet is urinating a normal amount	2	5	4.7	0.8
Mobility	My pet moves normally	2	5	4.5	0.9
	My pet lays in one place all day long	1	5	1.8	1.2
	My pet is active as it has been	1	5	4.3	1.1

The majority of the owners assessed their dogs’ mental state as good and their lives as happy. Most of the owners did not feel their dogs were showing signs of pain after the treatment and in their owners’ opinion, the dogs’ appetite and water intake were unchanged and they did not feel nauseated, vomited or have diarrhoea. Most of the owners did not notice hygiene or mobility disorders in their dogs.

The four additional questions were related to wound care and treatment costs. The results showed that 75% of the owners took care of their dog's wound, while 16% did not or the wound care was performed by another family member (4.5%). Two owners (4.5%) did not provide the information. To make it easier to take care of the wound, some of the owners expressed their desire for some additional material (25% written, 13.6% image and 2.3% video material) or a presentation of wound care by an expert (27.3%). The majority of the dogs' owners (86.4%) would opt for the therapy again, regardless of the financial costs. Three owners did not provide this information.

Overall, the owners assessed the general health compared with the initial diagnosis of cancer (from 1–worse to 5–better) as improving (mean 3.9, *SD* 1.1). In addition, the owners assessed their dogs' current HRQoL as good (from 1–very poor to 10–excellent) (mean 7.4, *SD* 2.8).

Specifically, the assessment of the dogs' current HRQoL compared the groups of dogs according to the treatment type (invasive vs. non‐invasive treatment group), treatment response (Nguyen et al., 2015) (objective response (OR) vs. progressive disease (PD)), tumour size (tumour volume < 3 cm^3^ or > 3 cm^3^) and tumour type (oral vs. cutaneous and subcutaneous). The results are shown in Table 3.

**Table 3:** Descriptive statistics for the independent variables (type of treatment, treatment outcome, type of tumour and tumour size) and the dogs’ current HRQoL assessment

	*n*	Mean	*SD*	Min	Max
Type of treatment					
Non‐invasive (*ECT + GET, ECT, GET*)	33	8.07	2.016	1	10
Invasive (*Surgery + ECT + GET, Surgery + ECT, Surgery + GET*)	11	5.11	3.723	1	10
Treatment outcome					
OR – objective response (CR – complete response, PR – partial response)	36	8.16	2.018	1	10
PD – progressive disease	8	3.86	3.078	1	8
Type of tumour					
Oral tumours	6	5.00	3.082	1	8
Other tumours (cutaneous and subcutaneous)	38	7.74	2.574	1	10
Tumour size					
>3 cm^3^	13	4.92	3.252	1	10
<3 cm^3^	26	8.62	1.329	6	10

Table 4 shows the differences among the treatment type, treatment outcome, type of tumour and tumour size and the dogs’ current HRQoL, assessed by the owners.

**Table 4:** Mann–Whitney U test for the independent variables (type of treatment, treatment outcome, type of tumour and tumour size) and the dogs’ current HRQoL assessment

	*M* (Current HRQoL†)	*N* ‡	*F*/*p* value
Type of treatment			
Non‐invasive (*ECT + GET, ECT, GET*)	8	30	73.000/*.035*
Invasive (*Surgery + ECT + GET, Surgery + ECT, Surgery + GET*)	5	9	
Treatment outcome			
OR – objective response (CR – complete response, PR –partial response)	8.5	32	26.000/*.001*
PD – progressive disease	3	7	
Type of tumour			
Oral tumours	5	5	37.000/*.040*
Other tumours (cutaneous and subcutaneous)	8	34	
Tumour size			
>3 cm^3^	5	13	52.500/*.000*
<3 cm^3^	9	26	

Abbreviation: M, median.

^†^ HRQoL Scored 1–very poor to 10–excellent.

^‡^ Five owners did not respond.

The findings show statistically significant differences (*p* < .05) between the type of treatment and the dogs’ current HRQoL. The owners assessed the current HRQoL as better among dogs receiving non‐invasive treatment types. Furthermore, the results show that the owners assessed the current HRQoL as better among dogs that had an OR to the treatment, compared to those that had PD despite the treatment (*p* < .05). HRQoL in dogs with oral tumours was assessed as worse than those receiving treatment for cutaneous and subcutaneous tumours (*p* < .05). Besides, the owners assessed the current HRQoL as better among dogs with smaller tumours (tumour volume below 3 cm^3^) than in those with larger tumours (tumour volume above 3 cm^3^) (*p* < .05).

## 4 DISCUSSION

Questionnaires assessing HRQoL are important tools for evaluating the effect of cancer and its treatment on body function and well‐being. Their use in veterinary medicine is becoming more and more important as they can help us with treatment decisions, provide prognostic information, evaluate the impact of novel treatments, improve the connection between owners and veterinarians and therefore make the owners feel more involved in the treatment procedure.

ECT is a local anti‐cancer treatment method that can be used in the management of various types of tumours in dogs (Kodre et al., 2009; Spugnini et al., 2013; Tozon et al., 2005). One of the main characteristics of this type of therapy is the low probability of systemic side effects and excellent post‐treatment function (e.g. mobility, food intake) of the treated animals (Kodre et al., 2009; Milevoj et al., 2019; Tozon et al., 2016; Tozon et al., 2005). When we combine it with IL‐12 GET, our aim is to complement the local effect of the treatment with the systemic anti‐cancer activity of IL‐12 (Cemazar et al., 2017; Cemazar et al., 2010; Cutrera et al., 2015; Reed et al., 2010). Both type of treatments can be combined with surgery. Surgery can be performed either as a neo‐adjuvant treatment to minimize the tumour burden, with ECT and/or GET performed subsequently after wound healing to treat microscopic residual disease. In cases of larger tumours or in less accessible locations, surgery can also be combined with intraoperative ECT and/or GET. When ECT and GET are coupled with surgery, prolonged wound healing is expected due to a more invasive treatment procedure.

**Figure 1:**
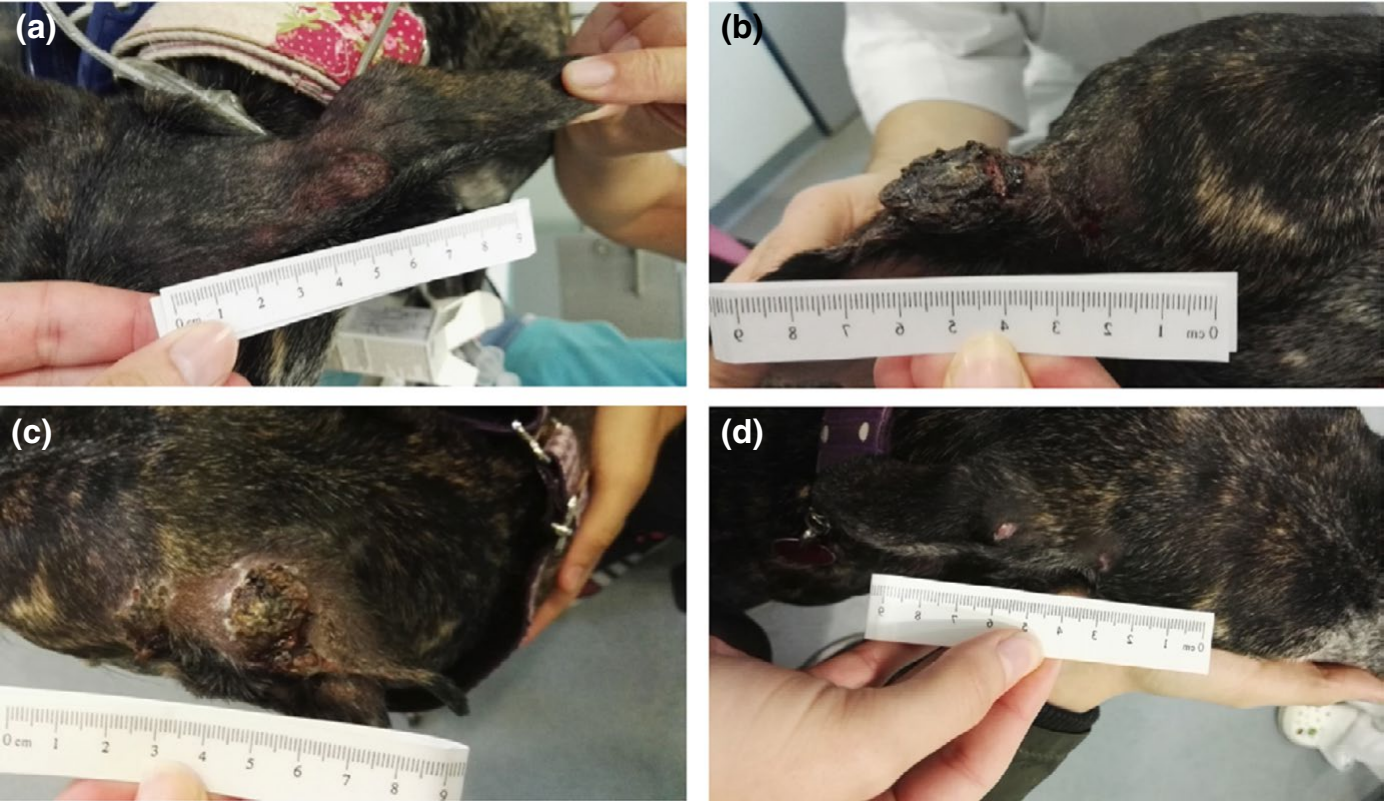
Treatment response to ECT and IL‐12 GET of a 6‐year‐old Greyhound with a mast cell tumour on the left ear base. (a) Before treatment, (b) 1 week after treatment (local inflammation with oedema and erythema), (c) 2 weeks after treatment (crust formation) and (d) 6 weeks after treatment (crust falls off and evaluation of treatment response can be performed)

After performing ECT and/or GET, tumour necrosis is possible and features such as oedema, erythema and local inflammation can last up to 2 weeks after treatment (Figure 1). After that, a crust is formed in the treated area and is expected to fall off approximately 1 month after treatment (Tozon et al., 2016). This is the optimal time to evaluate the response to the treatment according to the RECIST criteria (Nguyen et al., 2015) and the reason why the questionnaires were offered to the owners at least 1 month after treatment, when they have already experienced all the phases of wound healing. We believe that after the completion of treatment, the owners could also assess their dogs' HRQoL more objectively than during the period of tumour necrosis or wound healing.

The response to the treatment has an important effect not just on the treated animal, but also on their owner's perception on the HRQoL. It is natural that the owners of animals that have poor response to treatment will assess that the life quality of their animal is the same or even worse than after treatment, even when this is not obvious from the clinician's viewpoint. This may be an actual reflection of the owner's disappointment of the cancer treatment failure, which may be especially difficult to accept. It was already proven that the owners of non‐responder animals evaluated their animals' HRQoL as worse than the owners of responding animals (London et al., 2009). This was also shown in our study, where there was a statistically significant difference between the current HRQoL assessment between responders (dogs with an OR to treatment) and non‐responders (dogs with PD despite the treatment) (Table 3).

Whereas the response rate of cutaneous and subcutaneous tumours to ECT and/or IL‐12 GET is up to 100% (Cemazar et al., 2017; Kodre et al., 2009; Tozon et al., 2005), the response rate of oral tumours is still relatively low, ranging from 11% to 50%, depending on the tumour type (Cutrera et al., 2015; Milevoj et al., 2019; Reed et al., 2010). This is the reason we divided our treatment groups into dogs with oral tumours (oral malignant melanoma and oral fibrosarcoma) and compared the owner's perception of HRQoL with the rest of the dogs who had cutaneous and subcutaneous tumours of different histological diagnoses (Table 1). The owners of dogs with oral tumours evaluated their animals' HRQoL as statistically significantly worse than the owners of dogs with cutaneous and subcutaneous tumours.

Furthermore, we divided the dogs into invasive (when surgery was performed together with ECT and/or IL‐12 GET) and non‐invasive (ECT and/or IL‐12 GET only) treatment groups. We found that the owners of dogs in the non‐invasive treatment group assessed their dogs' HRQoL as significantly better than the ones in the invasive treatment group. The result could be explained to the fact that the healing process of wounds, resulting from electroporation‐based treatments combined with surgery, is expected to take more time. This can represent a bigger burden for the owner and may impact the dog's and the owner's life routine for a longer time than we expect if we do not combine ECT and/or IL‐12 GET with surgery. Some of the owners might also think that the HRQoL of those animals is impaired due to a need for more frequent visits to the veterinarian, which might represent a stress for some of the dogs. Also, a significant proportion of the dogs in the invasive treatment group were dogs that had oral tumours. Lower assessment of QoL in the invasive treatment group could therefore be assigned to a lower response rate rather than to the surgical procedure itself. This could potentially represent a bias of the results and a bigger cohort of patients with cutaneous and subcutaneous tumours, treated with ECT and/or GET in combination with surgery, could help us distinguish whether it is the treatment or the disease itself that has more significant impact on the dogs' QoL.

The size of a tumour correlates with the expected amount of necrosis after treatment with ECT and GET. Larger tumours can therefore represent a bigger burden for the owner because the treatment period is prolonged and the animals require more care from the owners. Also, in cases of larger tumours, a postoperative course of NSAIDs (3 to 5 days) is frequently required, which can influence the owners’ perception of QoL of the animals. This is why we compared the assessment of QoL of animals with smaller (tumour volume < 3 cm^3^) and larger (tumour volume > 3 cm^3^) tumours. The results indeed show that the owners assessed the current HRQoL as better among dogs with smaller tumours than in those with larger tumours.

The questionnaire also revealed a high owner compliance rate, with 80% of owners taking care of their animals’ wounds. Some of the owners expressed the desire for some additional written (25%), image (13.6%) or video (2.3%) material and a presentation of wound care by an expert (27.3%). This can be used as a guidance for further client education, meet the owners’ expectations regarding post‐treatment results and improvement of owner–veterinarian interaction.

There are three major limitations of the study. Firstly, in all of the instruments measuring HRQoL in animals, there is a need for proxy reporting, which is done by the owner. This may not necessarily represent the true HRQoL of the animal and can be influenced by several factors, for example, the personality of the owner or treatment outcome. The second limitation is that animals with oncological diseases are usually older and often have concurrent diseases that might impact their HRQoL. In those cases, it is often difficult for the owner to separate between the impact of the tumour or its treatment and the concurrent disease on their animal's life quality. The third limitation is that the questionnaire was designed for both dogs and cats that have very different behaviours and means of expressing their HRQoL. In the future, separate questionnaires should be designed for both species to obtain results that are more detailed. A minor limitation of the study is the internal reliability of the questionnaire, with the Cronbach *α* = 0.6712. Although Field (Field, 2009) states that the constructs values between 0.6 and 0.7 are the minimum acceptable value for Cronbach *α*, further psychometric analysis of the questionnaire should be performed.

The improvement in HRQoL and owner satisfaction with the treatment is in concordance with the results from the study performed in human patients, where the authors observed a significantly increased HRQoL in patients treated with ECT (Campana et al., 2016). In the mentioned study, the patient satisfaction with the treatment outcome was 80%, which is comparable to our data, demonstrating that 86% of owners were satisfied with the treatment and would opt for the therapy again, regardless of the cost. Moreover, the questionnaire has a wide valuable use not only for animals undergoing electroporation‐based treatments but also for other animals undergoing different oncological treatments (Lynch et al., 2011), and could be used in the future to evaluate HRQoL in bigger cohorts of cancer‐bearing animals. Namely, this is the first HRQoL questionnaire ever used in Slovenia and an important starting point for evaluation of HRQoL in Slovenian veterinary patients.

In conclusion, the results of this study provided a valuable and encouraging feedback from the owners of treated dogs. The questionnaire was very well accepted by the owners and proved as a useful tool for the assessment of HRQoL in dogs treated with ECT and/or IL‐12 GET. It could therefore serve as a tool for evaluating the HRQoL of animals undergoing other oncological treatments in the future. The owners of dogs with cutaneous and subcutaneous tumours treated with ECT and/or IL‐12 GET without addition of surgery and those that responded to the treatment evaluated their dogs' HRQoL as better than the owners of dogs with oral tumours, those where surgery was included in the treatment protocol and those that did not respond to the treatment.

## CONFLICT OF INTEREST

The authors declare no conflict of interest.

## ETHICAL STATEMENT

The authors confirm that the ethical policies of the journal, as noted on the journal's author guidelines page, have been adhered to. No ethical approval was required as this was a questionnaire‐based study that did not involve any direct work on experimental animals.

## Data Availability

The data that support the findings of this study are available from the corresponding author upon reasonable request.
